# The Influence of Movement Tempo During Resistance Training on Muscular Strength and Hypertrophy Responses: A Review

**DOI:** 10.1007/s40279-021-01465-2

**Published:** 2021-05-27

**Authors:** Michal Wilk, Adam Zajac, James J. Tufano

**Affiliations:** 1 Institute of Sport Sciences, The Jerzy Kukuczka Academy of Physical Education, Katowice, Poland; 2 Faculty of Physical Education and Sport, Charles University, Prague, Czech Republic

## Abstract

Hypertrophy and strength are two common long-term goals of resistance training that are mediated by the manipulation of numerous variables. One training variable that is often neglected but is essential to consider for achieving strength and hypertrophy gains is the movement tempo of particular repetitions. Although research has extensively investigated the effects of different intensities, volumes, and rest intervals on muscle growth, many of the present hypertrophy guidelines do not account for different movement tempos, likely only applying to volitional movement tempos. Changing the movement tempo during the eccentric and concentric phases can influence acute exercise variables, which form the basis for chronic adaptive changes to resistance training. To further elaborate on the already unclear anecdotal evidence of different movement tempos on muscle hypertrophy and strength development, one must acknowledge that the related scientific research does not provide equivocal evidence. Furthermore, there has been no assessment of the impact of duration of particular movement phases (eccentric vs. concentric) on chronic adaptations, making it difficult to draw definitive conclusions in terms of resistance-training recommendations. Therefore, the purpose of this review is to explain how variations in movement tempo can affect chronic adaptive changes. This article provides an overview of the available scientific data describing the impact of movement tempo on hypertrophy and strength development with a thorough analysis of changes in duration of particular phases of movement. Additionally, the review provides movement tempo-specific recommendations as well real training solutions for strength and conditioning coaches and athletes, depending on their goals.

## Key Points

**Table:** 

In addition to training loads, training volume, and rest intervals, movement tempo should be controlled and considered when planning and executing resistance-training programs.
Neither isolated slow nor fast movement tempos are more effective for muscle hypertrophy, but it seems that the most favorable is a combination of slower eccentric movements, paired with faster concentric movements.
Slower movement tempos require decreased external load, yet when paired with a greater time under tension, can create an adequate stimulus to induce hypertrophy and strength gains.
Faster concentric speeds are often thought to provide a better stimulus for neural adaptations and greater strength gains, but increasing the eccentric time under tension may help promote muscular hypertrophy, indirectly affecting strength without adversely affecting neural adaptations.

## Introduction

Hypertrophy and strength are two common long-term goals of resistance training, which are mediated by the manipulation of numerous variables including load, volume, exercise order, exercise selection, and the rest intervals between sets among others [1–4]. However, one training variable that is often neglected but is essential to consider for achieving these goals is the movement tempo of particular repetitions. Loosely speaking, there are two types of movement tempo during resistance training: unintentional and intentional. To elaborate, an unintentionally slow tempo can inadvertently occur during resistance training whereby a heavy load or the manifestation of fatigue is primarily responsible for a slower movement (i.e., increased duration of the repetition) [5]. Conversely, an intentional slow tempo can be purposefully used when the load is light enough to control and fatigue does not influence one’s ability to control the velocity of movement. Therefore, conscious and intentional control of the movement tempo is only possible to a certain extent [6, 7] during concentric actions where strength is a limiting factor, but is possible to a greater extent during eccentric actions where loads even above 100% of 1RM can be regulated to some degree. Regardless of whether the movement tempo is intentional or unintentional, tempo is often communicated using a sequence of digits (e.g., 2/0/3/0), where each digit defines the duration of a particular phase of the movement. According to the recommendations of a recent review on the topic, four-digit combination should be used, which describes the eccentric, isometric/transition, concentric, and isometric/transition phases [8]. For example, 2/0/3/0 denotes a 2-s eccentric phase, no intentional isometric pause during the transition phase, a 3-s concentric phase, and no pause between the completion of the concentric phase and the beginning (eccentric phase) of the next repetition. In this example, the movement tempo is intentional, as both the eccentric and concentric phases are performed at a certain cadence. However, if the digits were 2/0/*X*/0, the *X* represents a voluntary explosive action whereby the actual velocity and duration of the concentric phase is not controlled and may be involuntarily extended as fatigue manifests. Therefore, it is important to understand not only the prescription of different movement tempos, but also how intent and fatigue can result in different unintentional velocities.

Along these lines, acute changes in movement tempo affect the number of performed repetitions in a single set [9], the time under tension (TUT) [10], and the maximum possible load lifted during a resistance exercise [11–13]. Some studies have demonstrated that the number of possible repetitions decreases as the total intentional duration of each repetition increases when using the same load [10, 14]. Considering the relationship between movement tempo, the number of repetitions, and the TUT, they are not mutually exclusive, as they all affect each other [15, 16]. Thus, changing the movement tempo can indirectly cause the training load to be changed during a single training session, a training microcycle, or even a training mesocycle [13]. As a result, previous research has considered different movement tempos, loads, and number of repetitions, showing that changes in these variables affect physiological responses such as hormone and blood lactate concentrations [14, 17–19]. Furthermore, it has been suggested that increasing the duration of a given number of repetitions could increase the metabolic response during and after resistance training [19, 20], which is thought to be one of the driving factors for increasing strength and muscle hypertrophy [21]. Additionally, it has been postulated that intentionally slowing down the movement tempo reduces the momentum in a lift, thereby increasing the muscle activity which could positively mediate intracellular anabolic signaling, also promoting a greater hypertrophic response [22]. Therefore, it is critical to understand how changing movement tempo not only directly affects individual repetitions but also whether these minor changes can amount to greater chronic strength and hypertrophy adaptations.

Although a recent review has helped to shed light on the effects of movement tempo on acute exercise variables, the direct impact of movement tempo on acute responses forms the basis for chronic adaptive changes, which were not addressed in that review [8]. According to the American College of Sports Medicine [23], untrained individuals should use slow and moderate movement tempos [24–29]. For intermediate training, it is recommended that moderate tempos should be used [24–29], and for advanced athletes, a variety of tempos from slow to fast velocities is recommended. Although many would agree with these general training guidelines, those recommendations did not account for the duration of particular movement phases (eccentric vs. concentric), thus making it difficult to draw definitive conclusions in terms of resistance-training recommendations. Therefore, the purpose of this review is to explain how variations in movement tempo can affect chronic strength and hypertrophy adaptations in response to isotonic (i.e., dynamic constant external resistance) resistance training (Table 1).

**Table 1:** Summary of studies exploring the influence of movement tempo during resistance training on muscular strength and hypertrophy responses

Reference	Tempo/load (%1RM or nRM)	Subjects	Duration (week)/frequency (days/week)	Protocol (exercise/sets/repetitions)	Main findings
Tanimoto and Ishii [18]	1/1/1/0 (50%1RM) 1/1/1/0 (80%1RM) 3/1/3/0 (50%1RM)	24 untrained men	12/3	Knee extension 3 sets × maximal number of repetitions 1RM in knee extension	↑ Muscle hypertrophy (CAS) between pre and post for 1/0/1/0 (80%1RM) ↑ Muscle hypertrophy (CAS) between pre and post for 3/0/3/0 (50%1RM) ↔ Muscle hypertrophy (CAS) between pre and post for 1/1/1/0 (50%1RM) ↑ Muscle hypertrophy (CAS) for 3/0/3/0 (50%1RM) compared to 1/1/1/0 (50%1RM) ↑ Muscle hypertrophy (CAS) for 1/1/1/0 (80%1RM) compared to 1/1/1/0 (50%1RM) ↑ Muscle strength (1RM) between pre and post for all tempos ↔ Muscle strength (1RM) between used tempos
Watanabe et al. [19]	1/0/1/0 (30%1RM) 3/1/3/0 (30%1RM)	35 untrained men and women	12/2	Knee extension and knee flexion 3 sets × 13 repetitions 1RM in knee extension and knee flexion	↑ Muscle hypertrophy (front muscle thickness) in 3/1/3/0 and 1/0/1/0 between pre to post training ↑ Muscle hypertrophy (front muscle thickness) in 3/1/3/0 compared to 1/0/1/0 ↑ Muscle hypertrophy (back muscle thickness) in 3/1/3/0 and 1/0/1/0 between pre to post training ↔ Muscle hypertrophy (back muscle thickness) in 3/1/3/0 compared to 1/0/1/0 ↔ Muscle strength in knee extension and knee flexion between 1/0/1/0 and 3/01/3/0
Keeler et al. [25]	5/0/10/0 (50%1RM) 4/0/2/0 (80%1RM)	14 untrained women	10/3	Leg extension, leg curl, leg press, bench press, compound Row, biceps curl, triceps extension, torso arm 1 set × 8–12 repetitions 1RM in all 8 exercise	↔ Muscle hypertrophy (lean mass) between pre and post for 5/0/10/0 ↔ Muscle hypertrophy (lean mass) between pre and post for 4/0/2/0 ↔ Muscle hypertrophy (lean mass) between 5/0/10/0 and 4/0/2/0 ↑ Muscle strength in all 8 exercise between pre and post for 5/0/10/0 ↑ Muscle strength in all 8 exercise between pre and post for 4/0/2/0 ↑ Muscle strength in leg extension, leg curl, leg press, bench press for 4/0/2/0 compared to 5/0/10/0
Morrissey et al. [27]	1/0/1/0 (8 RM) 2/0/2/0 (8 RM)	34 subject	7/3	Back squat 3 sets × 8 RM 1RM in back squat	↑ Muscle strength in back squat between pre and post for 1/0/1/0 ↑ Muscle strength in back squat between pre and post for 2/0/2/0 ↔ Muscle strength in back squat between 1/0/1/0 and 2/0/2/0
Munn et al. [28]	1/0/1/0 (6–8 RM) 2/0/2/0 (6–8 RM)	46 untrained men and women	7/3	Bicep curl 1 set × 6–8RM or 3 sets × 6–8RM 1RM in bicep curl	↑ Muscle strength in biceps curl between pre and post for 1/0/1/0 ↑ Muscle strength in biceps curl between pre and post for 2/0/2/0 ↑ Muscle strength in biceps curl for 1/0/1/0 compared to 2/0/2/0
Neils et al. [29]	5/0/10/0 (50%1RM) 4/0/2/0 (80%1RM)	16 men and women (3 months of experience)	8/3	Bench press, biceps curl, triceps extension, leg extension, leg curl, squat, upright row 1 set × 6–8 repetitions 1RM in bench press and squat	↔ Muscle hypertrophy (FFM) between pre and post for 4/0/2/0 ↔ Muscle hypertrophy (FFM) between pre and post for 5/0/10/0 ↑ Muscle strength in bench press and squat between pre and post for 5/0/10/0 ↑ Muscle strength in bench press and squat between pre and post for 4/0/2/0 ↔ Muscle strength in squat between 4/0/2/0 and 5/0/10/0 ↔ Muscle strength in bench press between 4/0/2/0 and 5/0/10/0
Westcott et al. [31]	4/0/2/1 4/0/10/0	65 untrained men and 82 untrained women	8–10/2–3	13 exercise 1 set × 8–12 repetitions for 4/0/2/1 and 4–6 repetitions for 4/0/10/0	↑ Muscle strength between pre and post for 4/0/2/1 ↑ Muscle strength between pre and post for 4/0/10/0 ↑ Muscle strength for 4/0/10/0 compared to 4/0/2/1
Tanimoto et al. [40]	1/0/1/0 (80–90%1RM) 3/0/1/0 (55–60%1RM)	9 trained men	13/2	Squat, bench press, latissimus dorsi pull-down, abdominal bend, back extension 1 set × maximal number of repetitions 1RM in all 5 exercise	↑ Muscle hypertrophy (muscle thickness) between pre and post for 1/0/1/0 ↑ Muscle hypertrophy (muscle thickness) between pre and post for 3/0/1/0 ↔ Muscle hypertrophy (muscle thickness) between 1/0/1/0 and 3/0/1/0 ↔ Muscle hypertrophy (DXA) between 1/0/1/0 and 3/0/1/0 ↑ Muscle hypertrophy (DXA) between pre and post for 1/0/1/0 ↑ Muscle hypertrophy (DXA) between pre and post for 3/0/1/0 ↑ Muscle strength (sum 1RM for 5 exercises) between pre and post for 3/0/1/0 ↑ Muscle strength (sum 1RM for 5 exercises) between pre and post for 1/0/1/0 ↔ Muscle strength (sum 1RM for 5 exercises) between 1/0/1/0 and 3/0/1/0 ↑ Muscle strength (back extension) for 1/0/1/0 compared to 3/0/1/0
Schuenke et al. [43]	2/0/1/0 (80–85%1RM) 4/0/10/0 (40–60%1RM)	34 untrained women	6/17	Squat, leg press, leg extension 3 sets × 6–10RM 1RM in squat, leg press, leg extension	↑ Muscle hypertrophy type fiber I, IIA, IIX (CSA) between pre and post for 2/0/1/0 ↑ Muscle hypertrophy type fiber IIA, IIX (CSA) between pre and post for 4/0/10/0 ↔ Muscle strength between 2/0/1/0 and 4/0/10/0
Gillies et al. [57]	6/0/2/0 (6–8RM) 2/0/6/0 (6–8RM)	28 trained women	9/3	Leg press, parallel squats, knee extensions and knee flexion 2 sets × 6–8 repetitions 1RM in leg press	↑ Muscle hypertrophy (both types I and IIA) between pre and post for 2/0/6/0 ↑ Muscle hypertrophy (type I) between pre and post for 6/0/2/0 ↑ Muscle strength in leg press between pre and post for 6/0/2/0 ↑ Muscle strength in leg press between pre and post for 2/0/6/0 ↔ Muscle strength in leg press between 2/0/6/0 and 6/0/2/0
Nogueira et al. [58]	3/0/1/0 (40,50 and 60%1RM) 3/0/3/0 (40, 50 and 60%1RM)	24 untrained men	10/2	Horizontal leg press, knee extension, knee flexion, chest press, seated row, elbow extension, elbow flexion 3 sets × 8–10 repetitions 1RM in bench press and leg press	↑ Muscle hypertrophy in rectus femoris (muscle thickness) between pre and post for 3/0/1/0 ↔ Muscle hypertrophy in rectus femoris (muscle thickness) between pre and post for 3/0/3/0 ↑ Muscle hypertrophy in biceps brachli (muscle thickness) between pre and post for 3/0/1/0 ↑ Muscle hypertrophy in biceps brachli (muscle thickness) between pre and post for 3/0/3/0 ↑ Muscle hypertrophy biceps brachli (muscle thickness) after 3/0/1/0 compared to 3/0/3/0 ↔ Muscle hypertrophy rectus femoris (muscle thickness) after 3/0/1/0 compared to 3/0/3/0 ↑ Muscle strength in bench press and leg press exercise between pre and post for 3/0/1/0 ↑ Muscle strength in bench press and leg press exercise between pre and post for 3/0/3/0 ↔ Muscle strength between 3/0/1/0 and 3/0/3/0 ↑ Muscle power in bench press and leg press exercise for 3/0/1/0 compared to 3/0/3/0 ↑ Muscle power in bench press and leg press exercise between pre and post for 3/0/1/0 ↑ Muscle power in bench press and leg press exercise between pre and post for 3/0/3/0
Pereira et al. [60]	4/0/1/0 (8RM) 1/0/1/0 (8RM)	12 untrained men	12/2	Scott curl exercise 3 sets × 8RM 1RM in scott curl	↑ Muscle hypertrophy (CSA) after 4/0/1/0 compared to pre-training ↔ Muscle hypertrophy (CSA) after 1/0/1/0 compared to pre ↑ Muscle hypertrophy (CSA) after 4/0/1/0 compared 1/0/1/0 ↑ Muscle strength between pre and post in scott curl for 4/0/1/0 ↔ Muscle strength between pre and post in scott curl for 1/0/1/0 ↑ Muscle strength in scott curl after 4/0/1/0 compared 1/0/1/0
Bottaro et al. [75]	*X*/0/*X*/0 (60%1RM) 2–3/0/2–3/0 (60%1RM)	20 trained men	10/2	Leg press, knee extension, knee flexion, bench press, seated row, elbow extension, elbow flexion 3 sets × 8–10 repetitions 1RM in leg press, bench press	↑ Muscle strength in leg press and bench press between pre and post for *X*/0/*X*/0 ↑ Muscle strength in leg press and bench press between pre and post for 2–3/0/2–3/0 ↔ Muscle strength in leg press and bench press between *X*/0/*X*/0 and 2–3/0/2–3/0
Usui et al. [76]	1/0/1/1 (50%1RM) 3/0/3/0 (50%1RM)	16 untrained men	3/8	Parallel squat 3 sets × 10 repetitions 1RM in parallel squat	↑ Muscle hypertrophy in quadriceps femoris (muscle thickness) between pre and post for 3/0/3/0 ↔ Muscle hypertrophy in quadriceps femoris (muscle thickness) between pre and post for 1/0/1/0 ↑ Muscle strength in squat between pre and post for 3/0/3/0 ↔ Muscle strength in squat between pre and post for 1/0/1/0
Watanabe et al. [77]	1/0/1/0 (50%1RM) 3/1/3/0 (50%1RM)	18 untrained men and women	12/2	Knee extension, knee flexion 3 sets × 8 repetitions 1RM in knee extension, knee flexion	↑ Muscle hypertrophy (CSA) in 3/1/3/0 between pre to post training ↔ Muscle hypertrophy (CSA) in 1/0/1/0 between pre to post training ↑ Muscle hypertrophy (CSA) for 3/1/3/0 compared to 1/0/1/0 ↑ Muscle strength in knee extension and knee flexion between pre and post for 3/0/3/0 ↑ Muscle strength in knee extension and knee flexion between pre and post for 1/0/1/0 ↔ Muscle strength between tempo 1/0/1/0 compared to 3/0/3/0
Fielding et al. [87]	2/1/*X*/0 (70%1RM) 2/1/2/0 (70%1RM)	30 untrained men	16/3	Leg press, knee extension 3 sets × 8 repetitions 1RM in leg press, knee extension	↑ Muscle strength in leg press and knee extension between pre and post for 2/1/*X*/0 ↑ Muscle strength in leg press and knee extension between pre and post for 2/1/2/0 ↔ Muscle strength in leg press and knee extension 2/1/*X*/0 and 2/1/2/0

*Tempo of movement* eccentric/isometric/concentric/isometric, *1RM* one repetition maximum, *CSA* cross-sectional area, *FFM* free fat mass, *DXA* dual-energy X-ray absorptiometry, ↑ denotes significant increases, ↔ denotes no significant differences, ↓ denotes significant decreases

## Literature Search

Google Scholar, MEDLINE, SCOPUS, ADONIS, ERIC, SPORTDiscus, EBSCOhost, and PubMed databases were searched for all studies investigating the tempo of movement. The search was performed using the following keyword combinations: (‘tempo of movement’ OR ‘velocity of movement’ OR ‘repetition duration’ OR ‘speed of movement’ OR ‘time under tension’ OR ‘eccentric duration’ OR ‘concentric duration’ OR ‘number of repetitions’) AND (‘hypertrophy’ OR ‘muscle mass’ OR ‘strength’ OR ‘resistance exercise’ OR ‘performance’). The present review includes studies that [1] presented original research data on healthy adult participants, [2] precise determination of movement tempo was used, [3] were published in peer-reviewed journals, [4] were published in the English language, and [5] used isotonic resistance exercises during the experimental procedures. The literature search was conducted in March 2020 and included articles that were published from 1985 to March 2020.

## Influence of Movement Tempo on Muscle Hypertrophy

It has been well established that regular resistance training is an effective means to increase skeletal muscle mass, and findings from previous research suggest that a wide range of movement tempos can be used during resistance training to stimulate muscular hypertrophy [22]. Evidence suggests that intentionally slowing down the movement tempo of a single repetition increases the TUT and can increase muscle activation for a given number of repetitions [8, 30]. Hypothetically, increasing the activity of the muscle combined with longer TUT could positively mediate intracellular anabolic signaling, promoting a greater hypertrophic response [22]. However, the anabolic process is a complex phenomenon, and a change in one variable (e.g., tempo) can directly or indirectly affect other variables that also play a significant role in anabolic processes (e.g., load, number of performed repetitions, TUT, fatigue, etc.) [8]. Thus, increasing the TUT, combined with other neuromuscular and metabolic factors, could reduce not only the total number of possible repetitions, but also the dynamic inertia during a lift. Subsequently, less dynamic inertia likely requires less force output during the eccentric–concentric transition and during the concentric phase [8, 22, 31, 32]. As a result, performing fewer repetitions with lower force requirements may diminish any positive effects that increasing individual TUT and muscular activity may provide. Therefore, skeletal muscle growth could be positively or negatively affected by changing the movement tempo, depending on a myriad of other interrelated factors.

Although research has extensively investigated the effects of different intensities, volumes, and rest intervals on muscle growth [1–4], many of the present hypertrophy guidelines do not account for different movement tempos, likely only applying to volitional movement tempo. To further elaborate on the already unclear anecdotal evidence of different movement tempos on muscle hypertrophy, the research on the topic does not provide equivocal evidence. As previously mentioned, the ACSM [23] recommends a moderate or slower tempo of movement for novice- and intermediate-trained individuals, but a combination of slow, moderate, and fast tempos for advanced training, depending on the load and the repetition number [23, 28, 33]. Regarding hypertrophy, these guidelines generally concur with one fairly recent meta-analysis [22] which indicates that similar hypertrophic responses occur when the repetition duration ranges from 0.5 to 8 s, which is a very wide range, whereby acute exercise stress could largely vary [8]. However, it must be noted that neither the meta-analysis [22] nor the ACSM [23] recommendations accounted for the duration of particular movement phases (eccentric vs. concentric), thus making it difficult to draw definitive conclusions in terms of resistance-training recommendations. Considering the favorable hypertrophic effects of traditional eccentric exercise [6, 7, 34–37] compared against the favorable hypertrophic findings of traditional concentric exercise [38, 39] (i.e., normal dynamic constant external resistance training, not accompanied by blood flow restriction, etc.), it is important to differentiate concentric and eccentric durations during different movement tempos under standard resistance-training conditions.

To determine the impact of movement tempo with changes in the duration of both the eccentric and concentric phases on muscular hypertrophy, Tanimoto and Ishii [18] compared training programs with medium tempo (MED) with a light load (3/1/3/0; 50%1RM), fast tempo (FAS) with a heavy load (1/1/1/0; 80%1RM), and FAS tempo with a lighter load (1/1/1/0; 50%1RM) [18]. All three protocols included 3 sets of knee extensions performed to muscular fatigue with 60-s rest intervals for a period of 12 weeks. When using the same load (50%1RM), the MED tempo resulted in significantly greater hypertrophy based on cross-sectional area (CSA) than the FAS tempo. Since the intensity and the amount of total work were the same for the MED and FAS tempos, the slower tempo during MED can be considered as the primary factor responsible for the greater hypertrophic effect of the MED tempo. However, when comparing the effect of training with a MED tempo with a lighter load (50%1RM) to the FAS tempo with a heavier load (80%1RM), there was no difference in the hypertrophic response. Therefore, the study by Tanimoto and Ishii [18] indicates that slowing down the tempo may compensate for decreasing the load used when the training goal is hypertrophy. However, it should be noted that event if the same tempo is used, lighter loads can still induce similar hypertrophic responses as heavier loads as long as there is sufficient muscle fatigue [2]. Nevertheless, the results of Tanimoto and Ishii [18] showed that a constant load of 50%1RM with MED tempo resulted in greater hypertrophy than the FAS tempo. However, this study compared the effect of a single resistance exercise, which may not necessarily translate into a whole-body training program. To determine the effects of movement tempo during a whole-body resistance-training program (squat, chest press, latissimus dorsi pull-down, abdominal crunches, and back extensions), Tanimoto et al. [40] investigated MED (3/0/3/0; 55–60%1RM) and FAS (1/0/1/1; 80–90%1RM) tempos over 13 weeks using 3 sets of repetitions performed to muscular fatigue and 60-s rest intervals. They showed that despite using a lighter load with a MED tempo, similar hypertrophic effects were observed compared to the FAS tempo with a heavier load, which is consistent with the single-joint individual exercise results presented previously [18].

Although the load, or the mechanical stimulus, has been suggested to be of critical importance for inducing hypertrophic adaptations [36, 41, 42], most studies use different external loads for the faster and slower tempos [18, 25, 40, 43], which results in different mechanical stimuli. Furthermore, the meta-analysis made by Schoenfeld et al. [2] showed that not only the external load but also the point of muscle failure during the set is an important factor affecting hypertrophy. Therefore, the results of the two previously described studies [18, 40] indicate that movement tempo is an important variable that also plays a significant role in the anabolic process, and slower movements can be useful to compensate for any decreases in the load used as long as the exercises are performed to muscular failure. Although the data of Tanimoto and Ishii [18] and Tanimoto et al. [40] indicate that hypertrophy can be similar, or greater, with a MED movement tempo at 50–60%1RM compared to a FAS one at (80–90%1RM), not only differences in the load used, but also the volume of exercise should also be considered. Exercise volume is most often determined using the load and the number of repetitions, which can, at times, be overly simplistic [10, 30, 44].

When calculating training volume using sets*repetitions with the same load and exercise, a slower movement tempo decreases the maximum number of repetitions one is able to perform compared to a faster tempo [8, 9, 14, 45]. Thus, in these cases, a greater number of performed repetitions in a faster protocol, and thus volume, is not synonymous with greater TUT (i.e., the product of the number of repetitions and the TUT of each repetition). This becomes even more important to consider when factoring in the load used, as a single 3/0/3/0 repetition at 40%1RM may not result in the same stimulus as a 1.5/0/1.5/0 repetition at 80%1RM. In fact, multiple studies have shown that performing exercises using a slower tempo consisting of 5- or 6-s eccentric and concentric phases results in a significantly longer total TUT compared to a faster movement tempo [10, 14, 30, 46], but these studies did not consider the load, volume, and TUT all together. Since heavier loads result in greater motor unit recruitment and tension is one of the major stimulants of muscular hypertrophy and changes in muscle architecture [28], if the TUT is extended, greater hypertrophy adaptations could be achieved [22, 30]. Therefore, it can be assumed that the greater [40] or comparable [18] hypertrophy effect after a MED tempo with lighter load compared to a FAS tempo with heavier load in the aforementioned studies could have been partially associated with a greater total TUT.

Although longer TUT seems to play an important role in the hypertrophic responses, Schuenke et al. [43] reached partially contradictory findings. Schuenke et al. [43] found a statistically significant increase in total mean fiber area (CSA) after training (6–10RM; 3 sets; repetitions performed to muscular fatigue; 2-min rest interval; exercises: squat, leg press, leg extension; 2–3 times per week; 6 weeks) with a FAS (2/0/1/0; 80–85%1RM) when compared to slow (SLO) (4/0/10/0; 40–60%1RM) movement tempo (38.8% vs 10.6%; ES = 1.54 vs 0.65, respectively). The result of this study is contrary to others who suggest that a longer TUT may be beneficial for inducing muscle hypertrophy [30, 47]. However, according to Schoenfeld [48], hypertrophy occurs preferentially when the duration of the eccentric phase is increased; while in the study of Schuenke et al. [43], the duration of the concentric contraction was significantly extended.

It should be noted that limited evidence suggests that training at volitionally very slow durations (10 s per repetition) is superior or inferior from a hypertrophy standpoint, although a lack of controlled studies on the topic makes it difficult to draw definitive conclusions. One train of thought is that extremely slow tempos require lighter loads whereby the athlete can voluntarily control the tempo, which may be suboptimal for maximizing gains in muscle hypertrophy presumably as a result of inadequate motor unit recruitment and stimulation [22]. Furthermore, consideration should be given to determining not only the TUT for the entire movement but also independently for particular phases of the movement: TUT concentric (TUT-C) and TUT eccentric (TUT-E). Specifically, a slower eccentric movement increases the TUT-E, the level of metabolic stress, the hormonal responses [14, 46], and muscle fiber damage and protein degradation [49–51], inducing a stronger anabolic signal with the muscle and connective tissue. In contrast to a slower eccentric contraction, it seems beneficial to use faster concentric contractions for muscle hypertrophy by increasing muscle activation [52] and the rate of fatigue [53], which is more effective for stimulating the highest threshold motor units associated with type II fibers [43]. Folland and Williams [54] have suggested that these muscle fibers have a greater potential for muscle growth, even though this suggestion remains controversial [2, 55, 56]. This leads to the conclusion that not only is the TUT of the entire movement important to consider, but the relationship between the duration of the eccentric and concentric phases could also impact hypertrophy responses following resistance exercise.

Only one study compared resistance training using two conditions that were matched for total TUT (6/0/2/0 vs. 2/0/6/0), but with each condition including different TUT-C and TUT-E (9 weeks; 6–8RM repetitions performed to muscular fatigue; 2.5-min rest interval; lower body exercise: bilateral incline leg press, parallel squat, bilateral leg extension and leg extension; upper body exercise: bench press and one of three—bilateral bicep curl, lateral pull-down or seated row) [57]. The muscle biopsy analyses demonstrated that both type I and IIA vastus lateralis fiber areas significantly increased following the slower concentric contraction, while only type I fiber area increased following the slower eccentric contraction, but differences between groups were not significant. However, in a study by Gillies et al. [57], a simultaneous change in the duration of both the eccentric and concentric phases was induced. Under such conditions, it was impossible to accurately determine whether the beneficial effects of hypertrophy occurred as a result of changes in only the concentric duration, only the eccentric duration, or a combination of changes in both phases of movement.

The effects of extending only the duration of the concentric contraction on muscle hypertrophy were examined by Nogueira et al. [58] who showed that faster concentric contractions (3/0/1/0) resulted in greater muscle hypertrophy (CSA) compared to slower concentric contractions (3/0/3/0), but only in the biceps brachii, as there was no such effect in the rectus femoris (10 weeks; 20 training sessions; load: 40%1RM for the first two sessions, 50%1RM for the third and fourth sessions, and 60%1RM for the subsequent sessions; 8–10 repetitions; 90-s rest interval; exercise: horizontal leg press, knee extension, knee flexion, chest press, seated row, elbow extension, elbow flexion). The lack of hypertrophic benefits by extending the duration of the concentric phase has also been demonstrated in a study by Keeler et al. [25], where the authors compared resistance training (1 set; 8–12 repetitions to muscular fatigue; 60–90-s rest interval; exercises: leg press, leg curl, leg extension, anterior lateral pull-down, bench press, seated row, biceps curl, triceps extension) with a 5/0/10/0 tempo with 50%1RM, to 4/0/2/0 tempo performed at 80%1RM. However, their assessments were made in relation to fat-free mass (FFM), and although FFM measures provide a general estimate of hypertrophic gains over the course of a resistance-training program, they lack the sensitivity to evaluate subtle changes in muscle mass [59]. Compared to lengthening the concentric phase, a contrary effect was observed when the eccentric duration was extended in the study of Pereira et al. [60], where the participants performed 2 training sessions per week of the arm curl exercise, for 12 weeks with MED (4/0/1/0) or FAS (1/0/1/0) movement tempo. During each training session, 3 sets of 8RM were performed to muscular fatigue. The CSA in the biceps brachii was greater after training with the slower eccentric tempo compared to the faster one, which suggested that the extension of only the eccentric phase can be beneficial in the development of hypertrophy. However, more research should isolate the effects of eccentric durations to determine that these findings hold true in other exercises, populations, and eccentric durations.

Although this review focuses on the effects of different tempos during training, it is important to not overlook the fact that the 1RM procedures for the studies discussed above used volitional movement tempos. In fact, research has shown that slower tempos decrease the load lifted during 1RM tests when compared to faster tempos [11–13], meaning that if the same load is used during different tempos during training (based on a volitional tempo 1RM test), the intensity of the effort will not be the same and slower tempos increase the intensity of effort for a given load. Thus, the movement tempo used during training also impacts the rating of perceived exertion (RPE). Diniz et al. [61] demonstrated that strength training protocols matched for the number of sets and repetitions, load, and rest interval (3 sets; 6 repetitions; 60%1RM; 3-min rest intervals) but with different tempos (4/0/2/0, 2/0/2/0, *V*/0/*V*/0) (*V* represents volitional tempo of movement) produced different responses in RPE. Resistance training with a tempo of 4/0/2/0 yielded greater RPE compared to 2/0/2/0 and *V*/0/*V*/0. It is likely that due to the constrictive nature of muscle contractions, the physiological effect of slower movement tempo during resistance exercise can be similar to what occurs during resistance exercise with external occlusion in which the manipulation of blood flow restriction results in greater ratings of perceived exertion [62, 63]. Therefore, researchers and practitioners should consider how the tempo of the 1RM test can affect the subsequent program choices, as the principle of (testing) specificity reigns supreme when considering one’s relative maximum compared to their maximal performance abilities.

Another factor to consider when interpreting research is how changes in muscle tissue are assessed. Some authors assess regional specific hypertrophy and changes in fiber-type distribution via muscle biopsy [43, 64], while others have assessed changes in muscle CSA or thickness by either MRI or ultrasound [18, 40, 65]. Additionally, some studies indirectly assess site-specific changes in muscle growth and employ measures of overall fat-free mass (FFM) (i.e., DXA and densitometry) [25, 29, 66]. Although FFM measures provide a general estimate of hypertrophic gains over the course of a resistance-training program, they lack the sensitivity to assess subtle changes in muscle tissue [59]. In addition to skeletal muscle, FFM also includes such components as body fluids, bone, collagen, and other non-fatty tissues. Thus, it cannot be concluded that changes in FFM are specific to muscle hypertrophy.

Considering the data discussed throughout this section, movement tempo should be taken into consideration when planning and executing resistance-training programs to increase hypertrophy. Specifically, the results of studies indicate that neither isolated slow nor isolated fast movement tempos are effective for muscle hypertrophy, but it seems that the most favorable is a combination of slower movement in the eccentric phase with a faster movement during the concentric phase. However, the optimal use of variable movement tempos to increase muscle hypertrophy cannot be analyzed independently of other training variables (especially load, number of repetitions, TUT, etc.). Using a slower tempo during resistance exercises requires decreasing the external load compared to using a faster tempo [10–12], but can simultaneously increase the TUT during particular sets [3], possibly providing an adequate stimulus to induce hypertrophy [18, 40, 67], especially if the exercise is performed to muscle failure [2]. Therefore, if low-load resistance training is not carried out to muscular failure, high-load training appears to provide a superior hypertrophic stimulus, and thus greater growth of all muscle fibers [2]**.** However, exercise carried out to concentric failure with a longer TUT but a lighter load can be more effective in the development of hypertrophy than heavier loads with shorter TUT [40].

## Influence of Movement Tempo on Maximal Strength

Among other variables, movement tempo is an acute resistance-training variable that can be manipulated to potentially optimize maximal strength development. Strength improvements following resistance training tend to be most pronounced when the method of assessment is specific to the type of muscle action mode used in training [67], when heavier loads are used during training [68], and when the test is specific to the muscle actions trained [2, 69–72]. Compared to changes in muscle size, changes in strength appear to be largely dependent on the principle of specificity [71, 73]. For example, although one study showed that low-load (30%1RM) and high-load (80%1RM) resistance training resulted in similar changes in muscle growth, the heavier load resulted in greater changes in isotonic strength [55]. However, although the load used during training seems to affect strength adaptations, it is still exactly how faster or slower movement tempos influence strength gains. The possible advantages of faster tempos on strength were mentioned in a meta-analysis by Davies et al. [74], but the differences between strength gains following faster and slower tempos were not statistically significant. However, this meta-analysis, only compared fast or explosive movements to slower ones, and did not allow for accurate comparison between the concentric or eccentric phases of movement as well as between MED, SLO, or extremely slow (ESLO) tempos. Furthermore, the effects of movement tempo on strength, due to variations in the duration of the eccentric and concentric phases evaluated together may not give precise information about the impact of particular phases of movement (eccentric or concentric) on strength gains. Under such conditions, it is impossible to determine whether the potential benefits of varied tempo result from the changes in the duration of the eccentric or concentric contractions. Therefore, there is a need for a more accurate analysis of the impact of movement tempo on muscular strength.

Most of the research investigating the impact of different movement tempos on strength gains compared simultaneous changes in the duration of eccentric and concentric contractions, and most often in the range of 2–3-s variations [18, 19, 27, 28, 57, 75–77]. Although the duration of changes within 2–3 s is relatively small, it is particularly significant because even such differences may affect acute changes in the results of the 1RM test [12, 13], maximal number of repetitions performed [9, 10, 14], total TUT [10], and consequently, strength gains. Among the studies that compared the impact of movement tempo with 2–3-s changes of duration of particular phases, only one study by Munn et al. [28] showed that strength gains were greater after training (7 weeks, 1 set, 6–8RM performed to muscular fatigue, biceps curl; 2-min rest interval) with a 1/0/1/0 tempo compared to 2/0/2/0. However, during this study, single-joint resistance exercises were compared, and the strength increases were close to 2 kg, which is not practically relevant especially for experienced athletes. The remaining studies comparing the impact of simultaneous changes in duration (2–3 s) of the eccentric and concentric phases of movement did not show significant differences in strength gains between particular movement tempos [18, 19, 27, 28, 57, 75–77].

The lack of differences concerned trained and untrained subjects, training programs ranging from 3 to 13 weeks, and programs that consisted of single resistance exercises as well whole-body training programs. This led to the conclusion that relatively small changes in movement tempo do not affect strength gains. Furthermore, no significant differences in strength gains were observed despite some of these studies showing greater hypertrophy responses for MED compared to FAS tempos [19, 77]. However, it should be noted that all of these training programs contained a fairly high number of performed repetitions per set (from 6 to the maximal number of possible repetitions until failure) as well as load in the range from 30%1RM to 80%1RM, which does not necessarily represent optimal resistance-training practices for improving maximal dynamic strength [23, 78].

As most strength and conditioning professionals consider low-volume, heavy-load protocols to be ideal for developing maximal dynamic strength, it is again important to consider that such recommendations are primarily based on volitional movement tempos. As anecdotal evidence suggests that increasing the TUT can increase maximal strength, some researchers have investigated the effects of slow muscle actions and their effect on muscular strength. To begin, Schuenke et al. [43] did not show differences in strength gains after six weeks of resistance training (6–10RM performed to muscular fatigue; 3 sets; 2-min rest interval; exercises: squat, leg press, leg extension; 2–3 times per week; 6 weeks) with FAS (2/0/1/0 at 80–85%1RM) and SLO tempos (4/0/10/0 at 40–60%1RM). Similarly, a lack of significant differences in strength gains was observed by Neils et al. [29] who compared resistance training (1 set; 6–8 repetitions; exercise: bench press, biceps curl, triceps extension, leg extensions, leg curl, squat, upper right) with a MED movement tempo (4/0/2/0 at 80%1RM) to an ESL one (5/0/10/0 at 50%1RM). Both studies showed that large increases in the duration of movement tempo (9 s) did not impact strength gains. Surprisingly, the studies of Keeler et al. [25] and Neils et al. [29] used the same movement tempos, but reached contradictory results. Keeler et al. [25] showed that 10 weeks of resistance training (1 set, 8–12 repetitions performed to muscular fatigue; 60–90-s rest interval; exercises: leg press, leg curl, leg extension, anterior lateral pull-down, bench press, seated row, biceps curl, triceps extension) with a MED tempo (4/0/2/0) was more effective at improving strength compared to an ESL tempo (5/0/10/0). However, greater strength gains for ESL compared to MED movement tempo in the study of Keeler et al. [25] were not observed in each exercise. Therefore, the impact of movement tempo on strength may be related to the type of exercise used. Furthermore, untrained females were included in the study of Keeler et al. [25], while Neils et al. [29] used a mixed group of men and women with 3 months of resistance-training experience. The differences in both sex and training experience may have caused the conflicting results between the studies of Keeler et al. [25] and Neils et al. [29]. Additionally, the exercise protocols included differences not only in movement tempo, but also in the load used, which limits the possibility of assessing only the impact of movement tempo on muscular strength. Previous studies indicate that heavier loads (80–100%1RM) used during resistance training result in superior strength gains when compared to lower loads [24, 40]. Therefore, the differences not only in the tempo of movement but also in the load used can significantly affect the level of strength gains. Furthermore, the superior strength gains with the MED tempo compared to ESL in the study of Keeler et al. [25] occurred despite a significantly greater total TUT, especially the concentric TUT (~ 150 vs 60 s) in the group training with the ESL tempo, in each exercise and in all training sessions. However, it should be noted that ~ 150 s of resistance effort for a set is not optimal for strength gains, but may be preferable when developing strength endurance. For maximum strength gains, the ACSM [23] recommends that novice to intermediate individuals should use loads corresponding to 60–70%1RM for 8–12 repetitions (which translates to a total TUT of 24–60 s per set), for advanced individuals loads of 80–100%1RM for 1–6 repetitions (which translates to a total TUT of 3–25 s per set) to maximize muscular strength [23, 79–86]. This recommendation was not met in the study of Keeler et al. [25] in the group that used the ESL movement tempo, but was far surpassed (8–12 repetitions; tempo 5/0/10/0; TUT 120–180 s per set; 50%1RM).

Furthermore, much like the hypertrophy section of this review, the discrepancy in results of studies evaluating the impact of movement tempo on strength can be related to the fact that most of them consider simultaneous variations in the duration of both, the concentric and eccentric phases of movement. Under such circumstances, it is impossible to determine whether the potential strength gains occur as a result of changes in the duration of the eccentric or concentric phases of movement. Only four previous studies have compared the effects of changes in duration of only one phase of the movement on strength gains. Three of them compared the effect of changes in duration of the concentric contraction [31, 58, 87] and only one with changes in duration of the eccentric phase [60]. A study by Nogueira et al. [58] showed comparable strength gains after resistance training (10 weeks; 20 training sessions; load: 40%1RM for the first two sessions, 50%1RM for the third and fourth sessions, and 60%1RM for the subsequent sessions; 8–10 repetitions; 90-s rest interval; exercise: horizontal leg press, knee extension, knee flexion, chest press, seated row, elbow extension, elbow flexion) with faster and slower concentric tempos (3/0/1/0 vs. 3/0/3/0). Similar results were presented by Fielding et al. [87] who compared 2 training protocols (16 weeks, 3 sets, 8 reps; exercise: leg press, leg extension) with a 2/1/*X*/0 and 2/1/2/0 movement tempo (*X* represents maximum possible movement speed). However, in both of these studies, there were relatively small changes in the duration of concentric contractions which may not be sufficient to induce the differences in strength gains. The comparison of the impact of longer difference in the duration of concentric contractions was made by Westcott et al. [31] and it was shown that the slower concentric movement resulted in a greater increase in strength after 8–10 weeks of resistance training (1 set; exercise: leg extension, leg curl, leg press, neck flexion, neck extension, pullover, chest press, chest cross, lateral raise, bicep curl, triceps extension, abdominal crunch, low back) compared to the faster concentric movement (4–6 rep; 4/0/10/0 vs. 8–12 rep; 4/0/2/1). This result suggested that to induce significant strength gains between particular tempos, the differences in the duration of concentric contractions should be relatively high. However, it should be noted that in the study of Westcott et al. [31], increases in strength after resistance training with slower movement tempo were higher only by 3–4 kg, which may not be practically relevant, especially for experienced athletes. Further, from a practical point of view, the use of slower concentric movement and heavy external load (optimal for strength gains) seems impossible to implement especially in the training of advanced athletes and multi-joint exercises such as the squat. This is especially important when using high loads (90–100% of volitional 1RM), during which it is impossible to control and intentionally slow down the velocity during the concentric phase of movement. In addition, it should also be taken into account that increasing the duration of the repetitions also extends the duration of the training session. Considering the fact that most people who train consider the lack of time as a significant obstacle to training, the longer training duration may be unwarranted in certain situations [88].

Currently, only one previous study assessed the impact of changes in only the eccentric phase of movement during resistance exercise on strength gains [60]. This study showed that training (12 weeks, 2 times a week; 3 sets of 8 repetitions maximum, Scott curl exercise) with slower eccentric movement (4/0/1/0) was more effective at improving strength gains when compared to faster eccentric movement (1/0/1/0) [60]. The result of that study suggests that slower eccentric contractions with a simultaneous short duration of the concentric movement may be optimal for the development of muscular strength. The greater strength gains after training with slower eccentric contractions observed by Pereira et al. [60] can be related to prolonging TUT-E during each set [14]. The longer TUT-E increases metabolic stress and hormonal responses and muscle tension, which are thought to be important factors implicated in the promotion of muscular hypertrophy [30, 89], which can indirectly effect strength gains. Although increasing skeletal muscle size can serve as the basis for subsequent strength development, the study by Dankel et al. [72] showed that muscle hypertrophy did not contribute to the increase in strength and supports the notion that changes in strength are driven largely through the principle of specificity and neural adaptations. Further Mattocks et al. [71] showed that practicing the 1RM during training (i.e., high-load, low-volume training) increases strength to a similar degree as performing high-volume training whereby the high-volume training resulted in greater hypertrophy, while no changes were observed after the low-volume 1RM training. Furthermore, the strength improvement can by related to increases in muscle tension and altered fiber recruitment during longer eccentric duration of a resistance exercise [90]. Simultaneously the longer eccentric duration may actually limit the magnitude of eccentric force production, limiting the power output during the concentric contraction [31, 32], and decreasing the maximal load lifted [10–13] which could potentially limit the strength stimulus. Furthermore, the study by Pereira et al. [60] evaluated the effect of changing the duration of the eccentric contraction using only one single-joint exercise, which does not necessarily translate into multiple-joint exercises such as the squat or bench press. Therefore, the impact of manipulating the duration of only the eccentric contraction on strength gains requires further research.

Although interesting and scientifically sound studies have been done on the topic of movement tempo and changes in maximal strength, the current body of literature does not clearly indicate which tempo of movement is most effective for developing muscular strength. However, considering that to maximize muscular strength, loads of 80–100% of volitional 1RM are recommended, the concentric movement tempo not only depends on the athlete’s intent but mostly on the external load used. With greater loads decreasing maximal velocity in the concentric phase [91], the use of heavy loads over 85%1RM will necessitate a near-maximal or maximal effort to concentrically move the load, yet the actual concentric velocity of the lift will be relatively slow. Therefore, it seems that to maximize muscular strength, controlled slower movement tempos should be used only in the eccentric phase, which is possible even when the external load is more than 100%1RM [6, 7].

Faster resistance training is thought to provide a better stimulus for neural adaptations that lead to greater strength gains. On the other hand, some studies have demonstrated increased muscle activation with slower eccentric actions [30, 90], while others have shown the opposite, favoring faster eccentric actions (< 3 s) or no difference between the two phases of movement [90, 92, 93]. When looking beyond EMG studies to dynamic task-specific outcomes, the literature has shown that intentionally lengthening the eccentric duration may result in suboptimal strength adaptations when compared with faster eccentric actions [6, 7]. However, the increase in TUT-E by extending the duration of the eccentric contraction is thought to be an important factor for promoting muscular hypertrophy [30, 89] and indirectly affecting strength. Furthermore, in all of the aforementioned studies, the subjects that took part in the research had no experience in resistance training. Thus, the initial increase in strength is thought to be primarily from neural adaptations followed by larger contributions from muscle hypertrophy after the first few weeks of training [94]. In fact, Loenneke et al. [95] observed, using within subject correlational analysis, that changes in muscle size can explain up to 35% of the variance in muscle strength following an eight-week resistance-training program, which means that these results do not translate to athletes or people advanced in resistance training. Therefore, the impact of movement tempo on strength gains requires further studies, especially on athletes with heavy training loads, low number of repetitions, as well as short TUT.

## Conclusions

The results presented in this review indicate that changing the tempo of movement during resistance training may have an impact on the level of muscle hypertrophy and strength, however the results are not conclusive. The differences in the size of the muscles examined, the structure of the training programs, and the experimental approach used may all partially explain the discrepancy in results between the faster and slower tempos. Considering the data discussed throughout this review, movement tempo should be taken into consideration when planning and executing resistance-training programs to increase hypertrophy and strength. The results of studies indicate that neither isolated slow nor isolated fast movement tempos are more or less effective for muscle hypertrophy, but it seems that the most favorable is a combination of slower movement in the eccentric phase with a faster movement during the concentric phase. To increase strength, it is not clear whether any specific tempo is more effective than another. However, faster resistance training is thought to provide a better stimulus for neural adaptations, which could lead to greater strength gains. Lastly, it should be noted that there is a need for additional tempo-related investigation over longer training periods. Specifically, research should analyze the effects of changing individual phases (concentric or eccentric) of the movements. In doing so, it would be possible to better identify the importance of extending, or reducing, the eccentric or concentric phases.

## Limitations

While the total number of studies included in this review was relatively high, there is a lack of research representing particular populations such as the elderly, recreationally trained people, and competitive athletes. Specifically, only a small number of studies included highly trained participants, who would be the most likely candidates for advanced resistance-training methods like using different movement tempos. Considering the impact of movement tempo on muscular strength, there is no scientific data to clearly indicate the optimal training regimens for strength gains in heavy loaded multi-joint exercises, with a small number of repetitions, and relatively short TUT. Furthermore, deliberate manipulation of movement tempo may not be possible when exercises are performed with heavy loads or to muscular failure with fast concentric contractions. Furthermore, according to Wilk et al. [12, 13] during research or training, when a controlled movement tempo is used, the 1RM testing should be performed independently for particular movement tempos and the %1RM value should be determined upon the 1RM test trial at a specific movement tempo, what was not done in any of the studies presented in this review, which seems to be a significant limitation.

## Practical Implications

Considering that movement tempo is an important variable of resistance training, it should be precisely defined, especially within the scientific literature. Furthermore, training programs with variable movement tempos should determine the load (%RM) upon the 1RM test at a specific movement tempo [12, 13]. Additionally, TUT should be controlled. Furthermore, deliberate manipulation of movement tempo, may not be possible when an exercise is performed with heavy loads or to muscular failure with fast or extremely slow concentric contractions. Additionally, excessive attention at maintaining a certain movement tempo can negatively affect the quality of the exercise. Therefore, in practice, athletes should strive to maintain the optimal scope of duration of the eccentric and concentric contraction separately. Below, we propose a new classification related with the duration of particular phases of the movement (Table 2).

**Table 2:** Classification of scope of duration in particular movement phases for single contraction

Duration of a single contraction	Description	Abbreviation for contraction
*X*-maximal speed	Explosive contraction	EX
1–2.9 s	Fast contraction	F
3–5.9 s	Medium contraction	M
6–9.9 s	Slow contraction	S
10 s and above	Extremely slow contraction	ES

Although TUT is an indicator of training volume [10, 30, 96], and it is more and more often considered, currently there are no recommendations as to what value of TUT is optimal for the development of strength or muscle hypertrophy. The recommendations for strength development include a low number of repetitions 1–5 [23, 78] with volitional movement tempo, which translates approximately to 2–5 s of TUT for one repetition. Therefore, according to the collective consensus, the optimal TUT for strength gains should be between 2 and 20 s per set. For hypertrophy training, the optimal TUT for a set should be between 20 and 70 s (Table 3). However, the optimal TUT for specific training goals can also depend on the resistance-training experience, the type of exercise used, range of motion, as well as the level of fatigue during the sets and the entire training session.

**Table 3:** Optimal time under tension for specific training goals

Training goal	Number of repetitions (*n*)	Optimal time under tension for set (s)	Preferred duration in contraction (eccentric/concentric)
Strength	1–5	2–20	Fast/fast (F/F) Fast/explosive (F/EX) Medium/fast (M/F) Medium/explosive (M/EX)
Strength and hypertrophy	5–8	20–40	Fast/fast (F/F) Fast/explosive (F/EX) Medium/fast (M/F) Medium/medium (M/M) Slow/fast (S/F) Slow/medium (S/M)
Hypertrophy	8–12	40–70	Fast/fast (F/F) Fast/explosive (F/EX) Medium/fast (M/F) Medium/medium (M/M) Slow/fast (S/F) Slow/medium (S/M) Extreme slow/fast (ES/F) Extreme slow/medium (ES/M) Extreme slow/slow (ES/S) Extreme slow/extreme slow (ES/ES)

The changes in movement tempo impact the TUT in a single set as well as the TUT during a training session (exercise*set*rep*TUT; Table 4) [13]. However, there are currently no data available that determines the optimal TUT in a set for the development of muscle strength or hypertrophy; thus, this topic requires further research.

**Table 4:** The impact of different movement tempo on total time under tension

Exercise	Sets (*n*)	Reps (*n*)	Movement tempo	
			M/F 3/0/2/0	S/F 8/0/2/0
			TUT for exercise (s)	TUT for exercise (s)
Barbell squat	5	3	75	150
Barbell dead lift	4	3	60	120
One-leg barbell squat	4	6	120	240
Lying leg curls	4	6	120	240
Dumbbell lunges	2	8	80	160
Training time under tension			455	910

These examples show how the tempo of movement impacts training volume

*F* fast contraction, *M* medium contraction, *S* slow contraction, *ES* extremely slow contraction, *tempo of movement (3/0/2/0)* eccentric/isometric/concentric/isometric phase of each repetition

Furthermore, it must be stated that controlled movement tempos (e.g., 6/0/1/0) do not have to be used in every set or in every repetition [97], (Table 5). It can be speculated that alternating one set with a slower tempo (e.g., 5/0/5/0) and one with a faster one (e.g., 1/0/*X*/0) or performing “SLO/FAS super-sets” could combine the benefits of both, faster and slower movement tempos. Similarly, a variable tempo of movement can be used during a particular set of a resistance exercise, in which the first repetitions (e.g., reps 1–4) are performed at a faster tempo and then the following ones (e.g., reps 5–8) at a slower tempo. However, in doing so, the principle of specificity comes into play, and it should be acknowledged that alternating between faster and slower tempos may result in less focused adaptations. Furthermore, the tempo of movement is applicable not only in the programming of strength training, but also in relation to diagnostic tests that use external loads. Additionally, a variable tempo of movement can be favorable for youth and injured athletes, which for various health reasons are unable to use heavy loads, or perform explosive movements [8]. Furthermore, during slower movement tempos, it is easier to control particular phases of movement, which may be particularly beneficial in the recovery process of injured athletes [8].

**Table 5:** The real 13-week strength training program of the bench press exercise with different movement tempos of a professional powerlifter (World Champion)

	Set (*n*)	Rep (*n*)	Load (kg)	%1RM (%)	Tempo	Volume (rep × set)	TUT	TUT-E	TUT-C
**Session 1**									
Bench Press	6	6	140	61	2/0/*V*/0	36	108	72	~ 36
Training volume						36	108	72	~ 36
**Session 2**									
Bench Press	4	6	150	65	2/0/*V*/0	24	~ 72	48	~ 24
Bench Press	1	5	150	65	2/0/*V*/0	5	~ 15	10	~ 5
Training volume						31	~ 87	58	~ 29
**Session 3**									
Bench Press	6	6	140	65	6/0/2/0	36	288	216	72
Training volume						36	288	216	72
**Session 4**									
Bench Press	1	6	150	65	2/0/*V*/0	6	~ 18	12	~ 6
Bench Press	3	6	160	70	2/0/*V*/0	18	~ 54	36	~ 18
Bench Press	2	5	140	70	6/0/2/0	10	80	60	20
Training volume						34	~ 152	108	~ 44
**Session 5**									
Bench Press	3	6	160	70	2/0/2/0	18	72	36	36
Bench Press	3	4	160	70	5/0/5/0	12	120	60	60
Training volume						30	192	96	96
**Session 6**									
Bench Press	3	6	160	70	3/0/2/0	18	108	54	36
Training volume						18	108	54	36
**Session 7**									
Bench Press	3	5	170	74	2/0/*V*/0	15	~ 45	30	~ 15
Bench Press	2	4	170	74	2/0/*V*/0	8	~ 24	16	~ 8
Training volume						23	~ 69	46	~ 23
**Session 8**									
Bench Press	6	6	160	70	4/0/*X*/0	36	~ 162	144	~ 18
Training volume						36	~ 162	144	~ 18
**Session 9**									
Bench Press	1	6	160	70	2/0/*V*/0	6	~ 18	12	~ 6
Bench Press	1	5	170	74	2/0/*V*/0	5	~ 15	10	~ 5
Bench Press	1	4	180	78	2/0/*V*/0	4	~ 12	8	~ 4
Bench Press	1	6	160	70	3/0/*X*/0	6	~ 21	18	~ 3
Bench Press	1	5	170	74	3/0/*X*/0	5	~ 18	15	~ 3
Bench Press	1	4	180	78	3/0/*X*/0	4	~ 14	12	~ 2
Training volume						30	~ 98	75	~ 23
**Session 10**									
Bench Press	6	4	160	70	2/3/2/0	24	168	48	48
Training volume						24	168	48	48
**Session 11**									
Bench Press	2	6	160	70	*V*/1/*V*/0	12	~ 36	~ 12	~ 12
Training volume						12	~ 36	~ 12	~ 12
**Session 12**									
Bench Press	4	6	170	74	2/0/*V*/0	24	~ 72	48	~ 24
Bench Press	2	5	170	74	2/0/*V*/0	10	~ 30	20	~ 10
Training volume						34	~ 102	68	~ 34
**Session 13**									
Bench Press	2	5	170	74	2/0/*X*/0	10	~ 25	20	~ 5
Bench Press	2	5	180	78	2/0/*X*/0	10	~ 25	20	~ 5
Bench Press	2	5	170	74	3/0/*V*/0	10	~ 40	30	~ 10
Bench Press	2	4	180	78	3/0/*V*/0	8	~ 32	24	~ 8
Training volume						38	~ 122	94	~ 28
**Session 14**									
Bench Press	4	4	150	65	5/2/5/0	16	192	80	80
Training volume						16	192	80	80
**Session 15**									
Bench Press	4	5	180	78	*V*/0/*V*/0	20	~ 40	~ 20	~ 20
Training volume						20	~ 40	~ 20	~ 20
**Session 16**									
Bench Press	6	4	180	78	*V*/0/*V*/0	24	~ 48	~ 24	~ 24
Training volume						24	~ 48	~ 24	~ 24
**Session 17**									
Bench Press	6	5	180	78	2/0/2/0	30	120	60	60
Training volume						30	120	60	60
**Session 18**									
Bench Press	6	6	180	78	4/0/2/0	36	216	144	72
Training volume						36	216	144	72
**Session 19**									
Bench Press	4	4	190	83	*V*/0/*V*/0	16	~ 32	~ 16	~ 16
Training volume						16	~ 32	~ 16	~ 16
**Session 20**									
Bench Press	4	4	150	65	5/2/5/1	16	208	90	90
Training volume						16	208	90	90
**Session 21**									
Bench Press	3	5	190	83	*V*/0/*V*/0	15	~ 30	~ 15	~ 15
Bench Press	1	4	190	83	*V*/0/*X*/0	4	~ 6	~ 4	~ 2
Training volume						19	~ 36	~ 19	~ 17
**Session 22**									
Bench Press	4	3	200	87	*V*/0/*V*/0	12	~ 24	~ 12	~ 12
Training volume						12	~ 24	~ 12	~ 12
**Session 23**									
Bench Press	2	4	140	61	2/2/*V*/0	8	~ 40	16	~ 8
Bench Press	2	3	160	70	2/2/*V*/0	6	~ 30	12	~ 6
Bench Press	1	1	180	78	2/2/*V*/0	1	~ 5	2	~ 1
Bench Press	1	1	200	87	2/2/*V*/0	1	~ 5	2	1
Training volume						*16*	~ 80	32	~ 16
**Session 24**									
Bench Press	1	2	190	83	*V*/0/*V*/0	2	~ 4	~ 1	~ 1
Bench Press	2	2	200	87	*V*/0/*V*/0	4	~ 8	~ 4	~ 4
Bench Press	2	2	210	91	*V*/0/*V*/0	4	~ 8	~ 4	~ 4
Bench Press	1	2	220	96	*V*/0/*V*/0	2	~ 4	~ 2	~ 2
Bench Press	2	2	180	87	6/0/5/0	4	44	24	20
Training volume						16	~ 68	~ 35	~ 29
**Session 25**									
Bench Press	4	4	200	87	3/0/*V*/0	16	~ 64	39	~ 16
Bench Press	1	1	210	91	*V*/0/*V*/0	1	~ 2	~ 1	~ 1
Training volume						17	~ 66	~ 40	~ 17
**Session 26**									
Bench Press	1	4	180	78	*V*/0/*V*/0	4	~ 8	~ 4	~ 4
Bench Press	1	3	190	83	*V*/0/*V*/0	3	~ 6	~ 3	~ 3
Bench Press	1	2	200	87	*V*/0/*V*/0	2	~ 4	~ 2	~ 2
Bench Press	1	1	210	91	*V*/0/*V*/0	1	~ 2	~ 1	~ 1
Bench Press	1	1	220	96	*V*/0/*V*/0	1	~ 2	~ 1	~ 1
Bench Press	3	5	170	74	5/0/5/0	15	150	75	75
Training volume						26	~ 172	~ 86	~ 86
**Session 27**									
Bench Press	4	6	140	61	8/0/5/0	24	312	192	120
Training volume						24	312	192	120
**Session 28**									
Bench Press	2	4	180	78	*V*/0/*V*/0	8	~ 16	~ 8	~ 8
Bench Press	3	3	200	87	*V*/0/*V*/0	9	~ 18	~ 9	~ 9
Training volume						17	~ 34	~ 17	~ 17
**Session 29**									
Bench Press	1	3	180	78	*V*/0/*V*/0	3	~ 6	~ 3	~ 3
Bench Press	1	2	200	87	2/0/*V*/0	2	~ 6	4	~ 2
Bench Press	1	1	210	91	*V*/0/*V*/0	1	~ 2	~ 1	~ 1
Bench Press	1	1	220	96	*V*/0/*V*/0	1	~ 2	~ 1	~ 1
Bench Press	1	1	230	100	*V*/0/*V*/0	1	~ 2	~ 1	~ 1
Bench Press	1	1	210	91	8/0/*X*/0	1	~ 9	8	~ 1
Training volume						9	~ 27	~ 18	~ 9
**Session 30**									
Bench Press	3	4	140	61	5/0/5/0	12	120	60	60
Training volume						12	120	60	60
**Session 31**									
Bench Press	6	4	140	61	5/0/5/0	24	240	120	120
Training volume						24	240	120	120
**Session 32**									
Bench Press	6	2	200	87	2/1/2/1	12	72	24	24
Training volume						12	72	24	24
**Session 33**									
Bench Press	2	3	180	78	*V*/1/*V*/1	9	~ 36	~ 9	~ 9
Bench Press	2	2	200	87	*V*/1/*V*/1	4	~ 16	~ 4	~ 4
Bench Press	2	3	180	78	*V*/1/*V*/1	6	~ 24	~ 6	~ 6
Bench Press	2	2	200	87	*V*/1/*V*/1	4	~ 16	~ 4	~ 4
Training volume						23	~ 92	~ 23	~ 23
**Session 34**									
Bench Press	6	4	140	61	8/1/2/1	24	264	192	48
Training volume						24	264	192	48
**Session 35**									
Bench Press	2	2	200	87	*V*/1/*V*/1	4	~ 16	~ 4	~ 4
Bench Press	2	2	210	91	*V*/1/*V*/1	4	~ 16	~ 4	~ 4
Bench Press	2	1	220	96	*V*/1/*V*/1	2	~ 8	~ 2	~ 2
Bench Press	1	2	230	100	*V*/1/*V*/1	2	~ 8	~ 2	~ 2
Bench Press	2	2	240	104	*V*/1/*V*/1	4	~ 16	~ 4	~ 4
Training volume						16	~ 64	~ 16	~ 16
**Session 36**									
Bench Press	2	4	200	87	*V*/1/*V*/1	8	~ 32	~ 8	~ 8
Bench Press	3	3	200	87	*V*/1/*V*/1	9	~ 36	~ 9	~ 9
Training volume						17	~ 68	~ 17	~ 17
**Session 37**									
Bench Press	6	4	140	61	6/1/*X*/1	24	~ 204	144	~ 12
Training volume						24	~ 204	144	~ 12
**Session 38**									
Bench Press	1	2	200	87	*V*/1/*V*/1	2	~ 8	~ 2	~ 2
Bench Press	2	2	210	91	*V*/1/*V*/1	4	~ 16	~ 4	~ 4
Bench Press	2	2	220	96	*V*/1/*V*/1	4	~ 16	~ 4	~ 4
Bench Press	1	1	230	100	*V*/1/*V*/1	1	~ 4	~ 1	~ 1
Bench Press	1	2	240	104	*V*/1/*V*/1	2	~ 8	~ 2	~ 2
Bench Press	1	1	245	106	*V*/1/*V*/1	1	~ 4	~ 1	~ 1
Training volume						14	~ 56	~ 14	~ 14
**Session 39**									
Bench Press	1	2	200	87	*V*/1/*V*/1	2	~ 8	~ 2	~ 2
Bench Press	1	1	210	91	*V*/1/*V*/1	1	~ 4	~ 1	~ 1
Bench Press	1	1	220	96	*V*/1/*V*/1	1	~ 4	~ 1	~ 1
Bench Press	1	1	230	100	*V*/1/*V*/1	1	~ 4	~ 1	~ 1
Training volume						5	~ 20	~ 5	~ 5
**Session 40**									
Bench Press	4	4	110	48	*X*/1/*X*/1	16	~ 48	~ 8	~ 8
Training volume						16	~ 48	~ 8	~ 8
**Competition at the World Bench Press Championships**									

Tempo of movement = eccentric/isometric/concentric/isometric. The assumption that *X* ~ 0.5 s; *V* ~ 1 s

*TUT* time under tension, *TUT-E* time under tension of eccentric contraction, *TUT-C* time under tension of concentric contraction

## References

[1] Schoenfeld BJ, Ogborn D, Krieger JW (2017). Dose-response relationship between weekly resistance training volume and increases in muscle mass: a systematic review and meta-analysis. J Sports Sci.

[2] Schoenfeld BJ, Grgic J, Ogborn D (2017). Strength and hypertrophy adaptations between low- vs. high-load resistance training: a systematic review and meta-analysis. J Strength Cond Res.

[3] Grgic J, Schoenfeld BJ, Skrepnik M (2018). Effects of rest interval duration in resistance training on measures of muscular strength: a systematic review. Sports Med.

[4] Nunes JP, Grgic J, Cunha PM (2020). What influence does resistance exercise order have on muscular strength gains and muscle hypertrophy? A systematic review and meta-analysis. Eur J Sport Sci.

[5] Mookerjee S, Ratamess NA (1999). Comparison of strength differences and joint action durations between full and partial range-of-motion bench press exercise. J Strength Cond Res.

[6] Suchomel TJ, Wagle JP, Douglas J (2019). Implementing eccentric resistance training—part 1: a brief review of existing methods. J Funct Morphol Kinesiol.

[7] Suchomel TJ, Wagle JP, Douglas J (2019). Implementing eccentric resistance training—part 2: practical recommendations. J Funct Morphol Kinesiol.

[8] Wilk M, Tufano JJ, Zajac A. The influence of movement tempo on acute neuromuscular, hormonal, and mechanical responses to resistance exercise—a mini-review. J Strength Cond Res. 2020;34(8):2369–83. 10.1519/JSC.0000000000003636. 10.1519/JSC.0000000000003636 32735429

[9] Sakamoto A, Sinclair P (2006). Effect of movement velocity on the relationship between training load and the number of repetitions of bench press. J Strength Cond Res.

[10] Wilk M, Gołaś A, Stastny P (2018). Does tempo of resistance exercise impact training volume?. J Hum Kinet.

[11] Headley SA, Henry K, Nindl BC (2011). Effects of lifting tempo on one repetition maximum and hormonal responses to a bench press protocol. J Strength Cond Res.

[12] Wilk M, Gepfert M, Krzysztofik M (2020). Impact of duration of eccentric movement in the one-repetition maximum test result in the bench press among women. J Sports Sci Med.

[13] Wilk M, Golas A, Zmijewski P (2020). The effects of the movement tempo on the one-repetition maximum bench press results. J Hum Kinet.

[14] Wilk M, Stastny P, Golas A (2018). Physiological responses to different neuromuscular movement task during eccentric bench press. Neuro Endocrinol Lett.

[15] Tran QT, Docherty D (2006). Dynamic training volume: a construct of both time under tension and volume load. J Sports Sci Med.

[16] Tran QT, Docherty D, Behm D (2006). The effects of varying time under tension and volume load on acute neuromuscular responses. Eur J Appl Physiol.

[17] Rogatzki MJ, Wright GA, Mikat RP (2014). Blood ammonium and lactate accumulation response to different training protocols using the parallel squat exercise. J Strength Cond Res.

[18] Tanimoto M, Ishii N (1985). Effects of low-intensity resistance exercise with slow movement and tonic force generation on muscular function in young men. J Appl Physiol.

[19] Watanabe Y, Tanimoto M, Ohgane A (2013). Increased muscle size and strength from slow-movement, low-intensity resistance exercise and tonic force generation. J Aging Phys Act.

[20] Migiano MJ, Vingren JL, Volek JS (2010). Endocrine response patterns to acute unilateral and bilateral resistance exercise in men. J Strength Cond Res.

[21] Carey Smith R, Rutherford OM (1995). The role of metabolites in strength training. I. A comparison of eccentric and concentric contractions. Eur J Appl Physiol.

[22] Schoenfeld BJ, Ogborn DI, Krieger JW (2015). Effect of repetition duration during resistance training on muscle hypertrophy: a systematic review and meta-analysis. Sports Med.

[23] American College of Sports Medicine (2009). American College of Sports Medicine position stand. Progression models in resistance training for healthy adults. Med Sci Sports Exerc.

[24] Hay JG, Andrews JG, Vaughan CL (1983). Effects of lifting rate on elbow torques exerted during arm curl exercises. Med Sci Sports Exerc.

[25] Keeler LK, Finkelstein LH, Miller W (2001). Early-phase adaptations of traditional-speed vs. superslow resistance training on strength and aerobic capacity in sedentary individuals. J Strength Cond Res.

[26] Lachance PF, Hortobagyi T (1994). Influence of cadence on muscular performance during push-up and pull-up exercises. J Strength Cond Res.

[27] Morrissey MC, Harman EA, Frykman PN (1998). Early phase differential effects of slow and fast barbell squat training. Am J Sports Med.

[28] Munn J, Herbert RD, Hancock MJ (2005). Resistance training for strength: effect of number of sets and contraction speed. Med Sci Sports Exerc.

[29] Neils CM, Udermann BE, Brice GA (2005). Influence of contraction velocity in untrained individuals over the initial early phase of resistance training. J Strength Cond Res.

[30] Burd NA, Andrews RJ, West DW (2012). Muscle time under tension during resistance exercise stimulates differential muscle protein sub-fractional synthetic responses in men. J Physiol.

[31] Westcott WL, Winett RA, Anderson ES (2001). Effects of regular and slow speed resistance training on muscle strength. J Sports Med Phys Fit.

[32] Wilk M, Golas A, Krzysztofik M (2019). The effects of eccentric cadence on power and velocity of the bar during the concentric phase of the bench press movement. J Sports Sci Med.

[33] Farthing JP, Chilibeck PD (2003). The effects of eccentric and concentric training at different velocities on muscle hypertrophy. Eur J Appl Physiol.

[34] O’Hagan FT, Sale DG, MacDougall JD (1995). Comparative effectiveness of accommodating and weight resistance training modes. Med Sci Sports Exerc.

[35] Higbie EJ, Cureton KJ, Warren GL (1996). Effects of concentric and eccentric training on muscle strength, cross-sectional area, and neural activation. J Appl Physiol.

[36] Hortobagyi T, Hill JP, Houmard JA (1996). Adaptive responses to muscle lengthening and shortening in humans. J Appl Physiol.

[37] Seger J, Arvidsson B, Thorstensson A (1998). Specific effects of eccentric and concentric training on muscle strength and morphology in humans. Eur J Appl Physiol.

[38] Carrasco D, Delp M, Chester AR (1999). Effect of concentric and eccentric muscle actions on muscle sympathetic nerve activity. J Appl Physiol.

[39] Hollander DB, Durand RJ, Trynicki JL (2003). RPE, pain, and physiological adjustment to concentric and eccentric contractions. Med Sci Sports Exerc.

[40] Tanimoto M, Sanada K, Yamamoto K (2008). Effects of whole-body low-intensity resistance training with slow movement and tonic force generation on muscular size and strength in young men. J Strength Cond Res.

[41] Dudley GA, Tesch PA, Harris MS (1991). Influence of eccentric actions on the metabolic cost of resistance exercise. Aviat Space Environ Med.

[42] McDonagh M, Davies C (1984). Adaptive responses of mammalian skeletal muscle to exercise with high loads. Eur J Appl Physiol.

[43] Schuenke MD, Herman JR, Gliders RM (2012). Early-phase muscular adaptations in response to slow-speed versus traditional resistance-training regimens. Eur J Appl Physiol.

[44] Goto K, Takahashi K, Yamamoto M (2008). Hormone and recovery responses to resistance exercise with slow movement. J Physiol Sci.

[45] Wilk M, Gepfert M, Krzysztofik M (2019). The influence of grip width on training volume during the bench press with different movement tempos. J Hum Kinet.

[46] Keogh JWL, Wilson GJ, Weatherby RP (1999). A cross-sectional comparison of different resistance training techniques in the bench press. J Strength Cond Res.

[47] Bird SP, Tarpenning KM, Marino FE (2005). Designing resistance training programmes to enhance muscular fitness: a review of the acute programme variables. Sports Med.

[48] Schoenfeld BJ (2010). The mechanisms of muscle hypertrophy and their application to resistance training. J Strength Cond Res.

[49] Fridén J, Sjostrom M, Ekblom B (1983). Myofibrillar damage following intense eccentric exercise in man. Int J Sports Med.

[50] Stauber WT, Clarkson PM, Fritz V (1990). Extracellular matrix disruption and pain after eccentric muscle action. J Appl Physiol.

[51] Enoka RM (1996). Eccentric contractions require unique activation strategies by the nervous system. J Appl Physiol (1985).

[52] Sakamoto A, Sinclair PJ (2012). Muscle activations under varying lifting speeds and intensities during bench press. Eur J Appl Physiol.

[53] Hoelting BD, Scheuermann BW, Barstow TJ (2001). Effect of contraction frequency on leg blood flow during knee extension exercise in humans. J Appl Physiol.

[54] Folland JP, Williams AG (2007). The adaptations to strength training : morphological and neurological contributions to increased strength. Sports Med.

[55] Mitchell CJ, Churchward-Venne TA, West DWD (2012). Resistance exercise load does not determine training-mediated hypertrophic gains in young men. J Appl Physiol (1985).

[56] Schoenfeld BJ, Vigotsky AD, Grgic J (2020). Do the anatomical and physiological properties of a muscle determine its adaptive response to different loading protocols?. Physiol Rep.

[57] Gillies EM, Putman CT, Bell GJ (2006). The effect of varying the time of concentric and eccentric muscle actions during resistance training on skeletal muscle adaptations in women. Eur J Appl Physiol.

[58] Nogueira W, Gentil P, Mello SN (2009). Effects of power training on muscle thickness of older men. Int J Sports Med.

[59] Levine JA, Abboud L, Barry M (2000). Measuring leg muscle and fat mass in humans: comparison of CT and dual-energy X-ray absorptiometry. J Appl Physiol.

[60] Pereira PEA, Motoyama YL, Esteves GJ (2016). Resistance training with slow speed of movement is better for hypertrophy and muscle strength gains than fast speed of movement. Int J Appl Exerc Physiol.

[61] Diniz RC, Martins-Costa HC, Machado SC (2014). Repetition duration influences ratings of perceived exertion [published correction appears in Percept Mot Skills. 2014;119(1):332]. Percept Mot Skills.

[62] Wilk M, Krzysztofik M, Gepfert M (2018). Technical and training related aspects of resistance training using blood flow restriction in competitive sport—a review. J Hum Kinet.

[63] Egan AD, Winchester JB, Foster C (2006). Using session RPE to monitor different methods of resistance exercise. J Sports Sci Med.

[64] Claflin DR, Larkin LM, Cederna PS (2011). Effects of high- and low-velocity resistance training on the contractile properties of skeletal muscle fibers from young and older humans. J Appl Physiol (1985).

[65] Young WB, Bilby GE (1993). The effect of voluntary effort to influence speed of contraction on strength, muscular power, and hypertrophy development. J Strength Cond Res.

[66] Rana SR, Chleboun GS, Gilders RM (2008). Comparison of early phase adaptations for traditional strength and endurance, and low velocity resistance training programs in college-aged women. J Strength Cond Res.

[67] Roig M, O'Brien K, Kirk G (2009). The effects of eccentric versus concentric resistance training on muscle strength and mass in healthy adults: a systematic review with meta-analysis. Br J Sports Med.

[68] English KL, Loehr JA, Lee SMC (2014). Early-phase musculoskeletal adaptations to different levels of eccentric resistance after 8 weeks of lower body training. Eur J Appl Physiol.

[69] Fisher J, Steele J, Smith D (2017). High- and low-load resistance training: interpretation and practical application of current research findings. Sport Med.

[70] Fisher J, Steele J, Androulakis-Korakakis P (2020). The strength-endurance continuum revisited: a critical commentary of the recommendation of different loading ranges for different muscular adaptations. J Trainol.

[71] Mattocks KT, Buckner AL, Jessee MB (2017). Practicing the test produces strength equivalent to higher volume training. Med Sci Sports Exerc.

[72] Dankel SJ, Counts BR, Barnett BE (2017). Muscle adaptations following 21 consecutive days of strength test familiarization compared with traditional training. Muscle Nerve.

[73] Buckner SL, Jessee MB, Mattocks KT (2017). Determining strength: a case for multiple methods of measurement. Sports Med.

[74] Davies T, Kuang K, Orr R (2017). Effect of movement velocity during resistance training on dynamic muscular strength: a systematic review and meta-analysis. Sports Med.

[75] Bottaro M, Machado SN, Nogueira W (2007). Effect of high versus low-velocity resistance training on muscular fitness and functional performance in older men. Eur J Appl Physiol.

[76] Usui S, Maeo S, Tayashiki K (2016). Low-load slow movement squat training increases muscle size and strength but not power. Int J Sports Med.

[77] Watanabe Y, Madarame H, Ogasawara R (2014). Effect of very low-intensity resistance training with slow movement on muscle size and strength in healthy older adults. Clin Physiol Funct Imaging.

[78] Baechle TR, Earle RW (2000). Essentials of strength training and conditioning.

[79] de Vos NJ, Singh NA, Ross DA (2008). Effect of power-training intensity on the contribution of force and velocity to peak power in older adults. J Aging Phys Act.

[80] Campos GE, Luecke TJ, Wendeln HK (2002). Muscular adaptations in response to three different resistance-training regimens: specificity of repetition maximum training zones. Eur J Appl Physiol.

[81] Hakkinen K, Alen M, Komi PV (1985). Changes in isometric force-and relaxation-time, electromyographic and muscle fiber characteristics of human skeletal muscle during strength training and detraining. Acta Physiol Scand.

[82] Peterson MD, Rhea MR, Alvar BA (2004). Maximizing strength development in athletes: a meta-analysis to determine the dose–response relationship. J Strength Cond Res.

[83] Rhea MR, Alvar BA, Burkett LN (2003). A meta-analysis to determine the dose response for strength development. Med Sci Sports Exerc.

[84] Rhea MR, Phillips WT, Burkett LN (2003). A comparison of linear and daily undulating periodized programs with equated volume and intensity for local muscular endurance. J Strength Cond Res.

[85] Stone WJ, Coulter SP (1994). Strength/endurance effects from three resistance training protocols with women. J Strength Cond Res.

[86] Weiss LW, Coney HD, Clark FC (1999). Differential functional adaptations to short-term low- moderate-, and high-repetition weight training. J Strength Cond Res.

[87] Fielding RA, LeBrasseur NK, Cuoco A (2002). High-velocity resistance training increases skeletal muscle peak power in older women. J Am Geriatr Soc.

[88] Krzysztofik M, Wilk M, Wojdała G (2019). Maximizing muscle hypertrophy: a systematic review of advanced resistance training techniques and methods. Int J Environ Res Public Health.

[89] Kubo K, Kanehisa H, Fukunaga T (2002). Effects of resistance and stretching training programmes on the viscoelastic properties of human tendon structures in vivo. J Physiol.

[90] Martins-Costa HC, Diniz RCR, Lima FV (2016). Longer repetition duration increases muscle activation and blood lactate response in matched resistance training protocols. Motriz: Revista de Educação Física.

[91] McArdle WD, Katch FI, Katch VL (2015). Exercise physiology: nutrition, energy and human performance.

[92] van den Tillaar R (2019). Effect of descent velocity upon muscle activation and performance in two-legged free weight back squats. Sports (Basel).

[93] Lacerda LT, Martins-Costa HC, Diniz RC (2016). Variations in repetition duration and repetition numbers influence muscular activation and blood lactate response in protocols equalized by time under tension. J Strength Cond Res.

[94] Moritani T, deVries HA (1979). Neural factors versus hypertrophy in the time course of muscle strength gain. Am J Phys Med.

[95] Loenneke JP, Rossow LM, Fahs CA (2017). Time-course of muscle growth, and its relationship with muscle strength in both young and older women. Geriatr Gerontol Int.

[96] Wilk M, Krzysztofik M, Maszczyk A (2019). The acute effects of caffeine intake on time under tension and power generated during the bench press movement. J Int Soc Sports Nutr.

[97] Wilk M, Jarosz J, Krzysztofik M, Filip-Stachnik A (2021). Contrast tempo of movement and its effect on power output and bar velocity during resistance exercise. Front Physiol.

